# 445. High HCV Treatment Success among People Living with HIV - Kyrgyzstan, 2018-2021

**DOI:** 10.1093/ofid/ofac492.520

**Published:** 2022-12-15

**Authors:** Nasyat Kemelbek Kyzy, Roberta Horth, Aida Karagulova, Sevak Alaverdyan, Gaukhar Mergenova, Dinara Otorbaeva, Dilyara Nabirova

**Affiliations:** Central Asia Field Epidemiology Training Program, Bishkek, Kyrgyzstan, Bishkek, Bishkek, Kyrgyzstan; US Centers for Disease Control and Prevention, Dulles, Virginia; City center of AIDS prevention and Control, Bishkek, Bishkek, Bishkek, Kyrgyzstan; American University of Armenia, Dushanbe, Yerevan, Armenia; Asfendiyarov Kazakh National Medical University, Almaty, Almaty, Kazakhstan; Department of Disease Prevention and State Sanitary and Epidemiological Supervision, Bishkek, Kyrgyzstan, Bishkek, Bishkek, Kyrgyzstan; US Centers for Disease Control and Prevention, Regional Office of Central Asia, Almaty, Kazakhstan, Almaty, Almaty, Kazakhstan

## Abstract

**Background:**

People living with HIV (PLHIV) with viral hepatitis C (HCV) co-infection have higher risk of liver failure and mortality. Globally, >2 million people globally have HIV/HCV co-infections. Central Asia is one of the regions most affected by HCV. Since 2018, Kyrgystan has provided HCV treatment free of charge to 427 PLHIV. We analyzed factors associated with HCV treatment success among PLHIV in Kyrgystan.

**Methods:**

We conducted retrospective cohort survey among PLHIV > 18 years old on HIV antiretroviral treatment that completed viral hepatitis C treatment before September 2021. We defined treatment success as having RNA PCR < 15 IU/mL post-treatment and negative DNA PCR 12 and 24 weeks post-treatment. We abstracted demographic, clinical, and laboratory data from the national registry and interviewed consenting participants using structured questionnaires. We used logistic regression to identify factors associated with treatment success.

**Results:**

Of 302 participants, 81% were ≥ 40 years old, 79% were male, 72% had ever injected drugs, 53% had ever been incarcerated, and 15% were on methadone. Treatment outcomes were classified as success among 86%. Reluctance to seek healthcare services due to fear of HIV status disclosure was reported by 39% of participants and was associated with reduced odds of treatment success (adjusted odds ratio [AOR]: 0.4, 95% CI: 0.2-1.0). Participants receiving SMS counseling (64%) had increased odds of treatment success (AOR: 2.4, 95% CI: 1.2-5.4) as did those who followed an HCV diet (AOR: 31, 95% CI: 1.4-6.8). Participants with an opportunistic infection (32%) and or HIV stage 3 or 4 (49%) also had decreased odds of treatment success (AOR: 0.3, 95% CI: 0.1-1.3; and AOR: 0.1, 95% CI: 0.01-0.5, respectively). Initiation of care in 2020 (COVID-19 pandemic beginning) was also associated with reduced odds (AOR: 0.3, 95% CI: 0.1-1.0).

**Figure:**
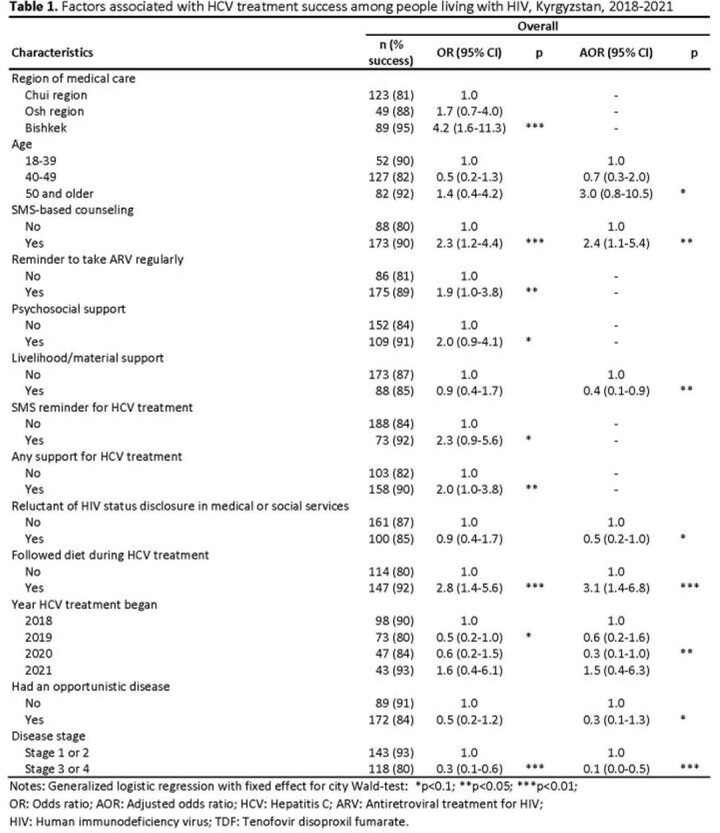

**Conclusion:**

Our study found high HCV treatment success in a difficult-to-treat population including during the COVID-19 pandemic. Programs that reduce stigma towards people living with HIV, promote early engagement in care, and provide SMS-based counseling could help increase treatment success for PLHIV in groups at higher risk for non-retention in care.

**Disclosures:**

**All Authors**: No reported disclosures.

